# Pertussis epidemic in Denmark, August 2023 to February 2024

**DOI:** 10.2807/1560-7917.ES.2024.29.14.2400160

**Published:** 2024-04-04

**Authors:** Anne Christine Nordholm, Hanne-Dorthe Emborg, Sarah Kristine Nørgaard, Ulrikka Nygaard, Aoife Ronayne, Lise Birk Nielsen, Bolette Søborg, Peter H Andersen, Tine Dalby

**Affiliations:** 1 Department of Infectious Disease Epidemiology and Prevention, Statens Serum Institut, Copenhagen, Denmark; 2 Department of Paediatrics and Adolescents, Rigshospitalet Copenhagen University Hospital, Copenhagen, Denmark; 3 Faculty of Health and Medical Sciences, University of Copenhagen, Copenhagen, Denmark; 4 Department of Bacteria, Parasites and Fungi, Statens Serum Institut, Copenhagen, Denmark

**Keywords:** Bordetella pertussis, whooping cough, epidemiology, surveillance, respiratory infection, public health

## Abstract

We report a record high pertussis epidemic in Denmark since August 2023. Highest incidence was in adolescents, while peak incidence in infants was lower vs previous epidemics in 2019 and 2016. Among infants aged 0–2 months, over half (29/48) were hospitalised and one infant died, underlining the disease severity in the youngest. To protect infants, pertussis vaccination in pregnant women was introduced in January 2024 in the national vaccination programme. Improved vaccination surveillance in pregnant women is being implemented.

Pertussis (whooping cough) is a highly contagious respiratory infection caused by *Bordetella pertussis*. The disease affects all ages, but infants (children < 1 year) experience the highest risk of severe disease and death [1]. From August 2023 to February 2024, there has been a pertussis epidemic in Denmark, which is described here with data up to 22 March 2024 from our national surveillance system. Relevant public health measures are highlighted.

## Surveillance of pertussis and vaccination coverage

Surveillance of infections and vaccination coverage is centrally administered at the national public health and research institute, Statens Serum Institut (SSI), under the Danish Ministry of Health. In Denmark, pertussis infections are laboratory notifiable, PCR is the primary diagnostic method, and information is recorded in the Danish Microbiology Database (MiBa), which is used for continuous surveillance [2]. We used the unique Danish personal identification number (CPR number) to link information on pertussis infections to information on demographics in the Civil Registration System to comorbidities and hospitalisations in the Danish National Patient Registry and to vaccinations in the Danish National Vaccination Registry. In Denmark, children are vaccinated with a combined vaccine providing immunisation against pertussis and four other severe diseases (diphtheria, tetanus, polio, and infections caused by *Haemophilus influenzae* type b) at 3, 5 and 12 months of age, and with a booster dose at 5 years of age.

## Post-COVID increase in pertussis infections

**Figure 1:**
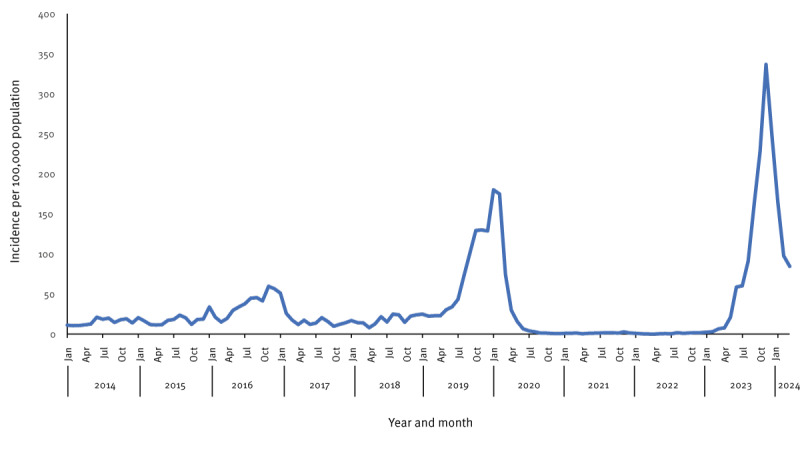
Pertussis incidence per year and month, Denmark, January 2014–March 2024 (n = 19,972 cases)

Pertussis has circulated with an interepidemic incidence of around 17 cases per 100,000 population per year in Denmark for the period 2014–22, except for the epidemic months in 2019 and 2016 (Figure 1). Though not strictly seasonal, pertussis has traditionally had a higher occurrence from August to November [3]. The most recent pertussis epidemic occurred in 2019–20, but nearly vanished from Denmark, along with other respiratory infections [4-7], following the non-pharmaceutical interventions implemented in 2020 to reduce COVID-19 transmission. In 2023, the number of pertussis cases increased remarkably (Figure 1).

Data from the national surveillance system hosted at Statens Serum Institut were extracted from the national microbiology database (MiBa) on 22 March 2024. The incidence was calculated by using population data from Statistics Denmark.

## Pertussis reaches epidemic levels

**Figure 2:**
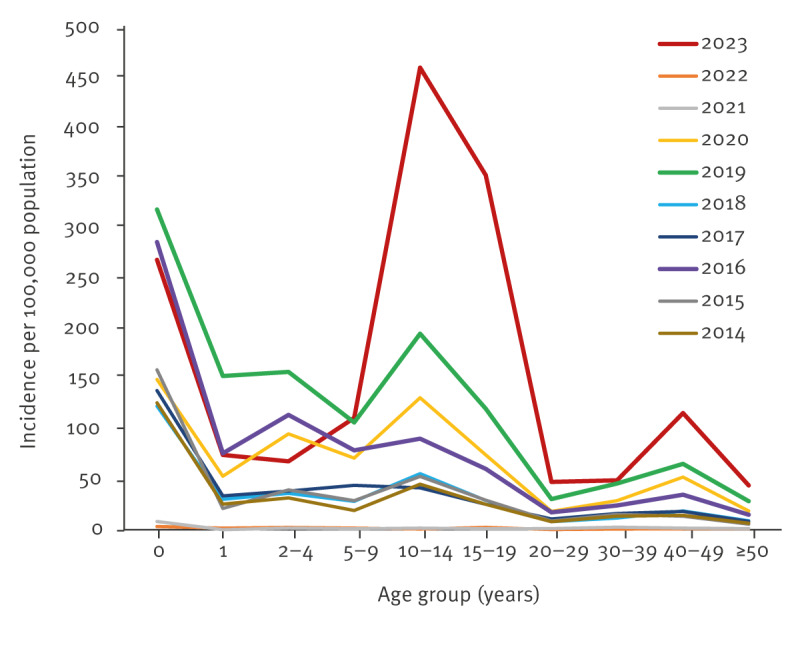
Pertussis incidence by age group and year, Denmark, 2014–2023 (n = 19,972 cases)

In Denmark, we define an epidemic level of pertussis as a more than three-fold increase in the incidence of pertussis (over interepidemic incidence levels) persisting at least 1 month and appearing in most of the country. In August 2023, the pertussis incidence increased to five times higher than the interepidemic incidence. The increase continued during the autumn, and the peak observed in November had an incidence of 337 cases per 100,000 population (Figure 1). The incidence declined in January and February 2024. The 2023 epidemic may be a manifestation of the natural cyclic nature of the disease, characterised by periodic epidemics every 3–5 years, but the number of cases was remarkably high. The age-specific incidences mirrored past epidemics, peaking in infants and adolescents (Figure 2), though the increase in adolescents reached a notably higher level. The infant incidence was lower in 2023 compared with previous epidemics (Figure 2).

**Figure 3:**
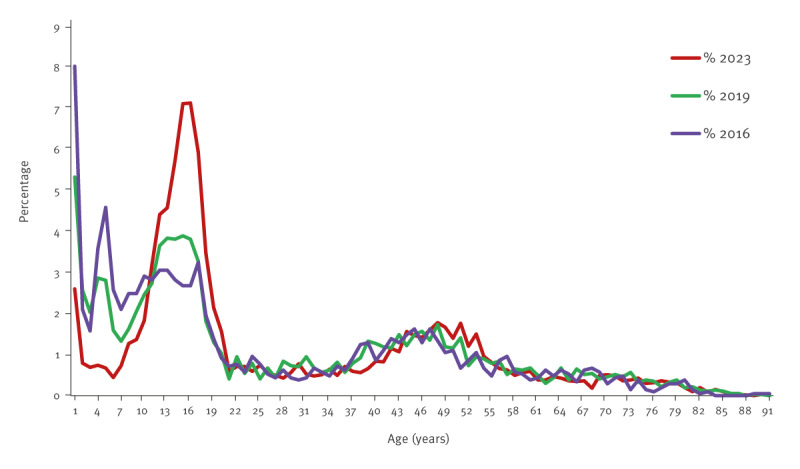
Percentage of pertussis cases by age, Denmark, epidemic years 2016, 2019 and 2023 (n = 11,841cases)

The proportion of infants (< 1 year) was 2.6% (158/6,061) in 2023, which is conspicuously lower than in 2019 (5.3%; 197/3,691) and 2016 (8.0%; 168/2,089) (Figure 3).

The number of cases per year: 2023 (n = 6,061), 2019 (n = 3,691) and 2016 (n = 2,089).

## Vaccination status in 2023

Among children under 2 years with pertussis in 2023, 37.9% (78/206) were unvaccinated, 48.1% (99/206) had received one or two vaccinations, and 14.1% (29/206) had received all vaccines before 2 years of age.

Approximately 85% of pregnant women in Denmark were vaccinated from August to December (week 31–52) in 2023. We had exact information on the number of vaccinations containing the combination of diphtheria + tetanus + pertussis (DTaP vaccinations) administered to adults during the 5-month period, and we used the number of newborns as a proxy for the number of pregnant women to get an estimate of the proportion of women vaccinated with DTaP during pregnancy. We did not have information on matching pairs of infants and mothers, but the number of newborns (0-year-olds at mid-2023) as a proxy for the number of pregnant women is feasible since the number of newborns in Denmark is relatively stable within a short timespan and thus can be used to estimate the number of pregnancies in that same period. From the reimbursement codes used by the general practitioner (GP), we had the exact number of DTaP vaccines that were administered in the period by week and region. There are no other adult boosters that are recommended in Denmark, and the majority of the pertussis vaccines administered to women of the childbearing age can be assumed to be related to pregnancy. The vaccination is recommended in late second or early third trimester, normally in conjunction with a pregnancy control visit at the GP in week 25 or 32.

## Pertussis incidence, disease severity and hospitalisations

In 2023, in total, 121 of 6,061 pertussis cases (2.0%) were hospitalised for more than 12 hours and most had no comorbidities (83.9%; 5,085/6,061). Among infants aged 0–2 months, 60.4% were hospitalised and one prematurely born infant whose mother was unvaccinated died. However, deaths among children with pertussis are rare in Denmark, with the last death reported in 2010 [8]. The proportion of infants 0–2 months of age who were hospitalised was markedly lower in 2023 (60.4%) than in 2019 (75.0%) and 2016 (78.8%) (Table), which may reflect protection against severe pertussis through vaccinated mothers. The overall incidence among infants was lower in 2023 compared with previous epidemics. The test-positive percentage was also lower among infants in 2023 compared with 2019 and 2016, but higher among adolescents (Table).

**Table:** Pertussis test positivity, hospitalisations and incidence, by age group, Denmark, epidemic years 2016, 2019 and 2023

Age group	Year	Number of tests	Individuals with a positive test	Test-positive percentage^a^	Individuals admitted to hospital^b^		Incidence per 100,000 population
		n	n	%	n	%	
0–2 months	2023	974	48	5.4	29	60.4	326.9
	2019	1,008	56	6.0	42	75.0	362.9
	2016	511	66	14.3	52	78.8	450.5
3–4 months	2023	645	53	9.0	20	37.7	541.4
	2019	701	50	7.4	31	62.0	486.0
	2016	373	43	11.8	36	83.7	440.2
5–11 months	2023	1,578	57	3.7	5	8.8	166.4
	2019	1,884	91	4.8	12	13.2	252.7
	2016	969	59	6.3	22	37.3	172.6
1–4 years	2023	5,734	176	3.1	8	4.5	69.9
	2019	5,887	380	6.6	11	2.9	156.2
	2016	2,865	248	8.8	12	4.8	105.0
5–9 years	2023	4,091	342	8.5	2	0.6	111.4
	2019	2,464	335	13.8	6	1.8	107.0
	2016	1,615	263	16.6	9	3.4	79.1
10–19 years	2023	10,433	2,748	26.5	14	0.5	405.4
	2019	5,487	1,078	20.1	7	0.6	157.7
	2016	2,225	515	23.6	22	4.3	75.3
≥ 20 years	2023	38,175	2,637	7.0	43	1.6	56.9
	2019	23,776	1,701	7.3	40	2.4	37.8
	2016	9,136	895	10.0	118	13.2	20.4

^a^ Test-positive percentage is calculated as the number of positive tests of the total number of tests. There are individuals with more than one positive test in the column ‘Number of tests’ as these numbers are not per individual whereas the other columns are restricted to individuals (no duplicates). The number of positive tests per year: 2023 (n = 6,061), 2019 (n = 3,691) and 2016 (n = 2,089).

^b^ Proportion admitted is calculated as the number of individuals admitted to the hospital of the number of individuals with a positive test.

## Discussion

Denmark experienced a record high pertussis epidemic from August 2023, peaking in November with an incidence of 337 cases per 100,000 population. The incidence slowly decreased in January and February 2024. The highest increase in incidence was among adolescents. The incidence peak in infants was lower than during the epidemics in 2019 and 2016. One prematurely born infant died, underlining the severity of pertussis in the youngest age group. The multi-country European Centre for Disease Prevention and Control (ECDC)-funded PERTINENT (Pertussis in Infants Network) study found that vaccination of women during pregnancy reduced hospitalisation in infants < 2 months of age by 75–88% [9]. In addition to a lower incidence among infants in Denmark during the 2023 epidemic, both the number and the proportion of infants admitted to the hospital was lower in 2023 compared with 2019 and 2016. This could reflect a vaccine effect as ca 85% of pregnant women were pertussis-vaccinated. 

On 1 January 2024, pertussis immunisation during pregnancy was endorsed by the Danish Health Authority as a permanent programme [10]. Vaccinations are registered in the Danish Vaccination Registry, but as information on pregnancy status is not entered, it is currently not possible to specifically survey vaccination coverage among pregnant women in Denmark. However, a rough estimate can be obtained via the billing codes specific for pertussis vaccination during pregnancy. To increase timely surveillance of vaccine uptake among pregnant women, an improved surveillance system is being implemented by SSI. A temporary vaccination programme for pregnant women – in place since November 2019 instigated as an outbreak mitigation measure – was continued until early 2023 but because of a low number of cases, a robust measure of vaccination effectiveness could not be obtained. Our current analysis has an extended study period through 2023 where the case load was very high.

The vaccination coverage among children during the COVID-19 pandemic has declined in many countries [11]. The resurgence of pertussis cases in Denmark, however, is not linked to a decline in immunisation rates, which have in fact increased in Denmark. The vaccination coverage is 97% for the first three vaccines in the childhood programme for the 2022 birth cohort and increased from 91% for the 2013 birth cohort [12]. The high case load reflects many susceptible individuals, which may be linked to the so-called ’immunity debt’ after COVID-19 [13]. A number of public health initiatives in Denmark aim to ensure timely childhood vaccinations with a proactive electronic reminder system, which reminds parents to schedule an appointment for vaccinations in the childhood vaccination programme. In addition to this, health visitors in Denmark act as ’vaccination ambassadors’ [14] as they, through several visits at the homes of newborns, introduce the childhood vaccination programme and may discuss any concerns or vaccine hesitancy with the parents.

Neither pertussis vaccination nor an episode of pertussis induces lasting immunity, and epidemics will occur regularly even in highly immunised populations [15,16]. A number of European countries have reported an increase in pertussis cases [17]. The high incidence among adolescents aged 10–19 years could be expected in Denmark, given there is no booster vaccination for this age group, although the magnitude in 2023 was nevertheless notable. Although the high number of cases could be attributed to increased awareness and testing practices, this explanation is not likely, as the positivity rate would have been lower compared with previous years. This consequently indicates a notable disease prevalence in adolescents. In the other Scandinavian countries, a pertussis booster vaccination is offered to adolescents [18,19]. In Denmark, the vaccination strategy does not aim to prevent all cases across all age groups. Instead, the programme focusses on mitigating pertussis-related infant morbidity and mortality by the newly implemented targeted vaccination during pregnancy and by encouraging timely administrations of childhood vaccinations.

## Conclusion

Here we report a record high pertussis epidemic in Denmark from August 2023 to February 2024, with the highest increase in incidence among adolescents. The incidence among infants was lower during the 2023 epidemic compared with previous epidemics, possibly as a result of pregnant women being vaccinated against pertussis. Overall, few were hospitalised, but among infants aged 0–2 months, 60% were hospitalised and one infant died, underlining the severity of the disease for this age group. Pertussis vaccination in pregnant women has recently become a permanent part of the national vaccination programme and improved surveillance of vaccinations in pregnant women is in the pipeline.
